# Accuracy of Pretransplant Imaging Diagnostic for Hepatocellular Carcinoma: A Retrospective German Multicenter Study

**DOI:** 10.1155/2019/8747438

**Published:** 2019-03-05

**Authors:** Uta Herden, Wenzel Schoening, Johann Pratschke, Steffen Manekeller, Andreas Paul, Richard Linke, Thomas Lorf, Frank Lehner, Felix Braun, Dirk L. Stippel, Robert Sucher, Hartmut Schmidt, Christian P. Strassburg, Markus Guba, Marieke van Rosmalen, Xavier Rogiers, Undine Samuel, Gerhard MSc Schön, Bjoern Nashan

**Affiliations:** ^1^Department of Hepatobiliary and Transplant Surgery, University Medical Center Hamburg-Eppendorf, Hamburg, Germany; ^2^Department of General, Visceral, and Transplantation Surgery, University Hospital of RWTH, Aachen, Germany; ^3^Department of Surgery, Campus Charité-Mitte and Campus Virchow-Klinikum, Charité, Berlin, Germany; ^4^Department of General, Visceral, Thoracic, and Vascular Surgery, University Hospital of Bonn, Bonn, Germany; ^5^Department of General, Visceral, and Transplantation Surgery, University of Duisburg-Essen, University Hospital Essen, Essen, Germany; ^6^Department of General and Visceral Surgery, Frankfurt University Hospital, Goethe-University Frankfurt/Main, Frankfurt/Main, Germany; ^7^Department of General, Visceral, and Transplant Surgery, University Medical Center Göttingen, Göttingen, Germany; ^8^Department of General, Visceral, and Transplantation Surgery, Hannover Medical School, Hannover, Germany; ^9^Department of General, Visceral, Thoracic, Transplantation, and Pediatric Surgery, University Medical Center Schleswig-Holstein, Kiel, Germany; ^10^Department of General, Visceral, and Cancer Surgery, University of Cologne, Köln, Germany; ^11^Department of Visceral, Transplantation, Vascular, and Thoracic Surgery, University Hospital of Leipzig, Leipzig, Germany; ^12^Department of Transplantation Medicine, University Hospital Münster, Münster, Germany; ^13^Eurotransplant Liver Intestine Advisory Committee, Eurotransplant International Foundation, Leiden, Netherlands; ^14^Eurotransplant International Foundation, Leiden, Netherlands; ^15^Department of Medical Biometry and Epidemiology, University Medical Center Hamburg-Eppendorf, Hamburg, Germany; ^16^Clinic for Hepatopancreaticobiliary Surgery and Transplantation, University of Science and Technology of China, Hefei, Anhui, China

## Abstract

Selection and prioritization of patients with HCC for LT are based on pretransplant imaging diagnostic, taking the risk of incorrect diagnosis. According to the German waitlist guidelines, imaging has to be reported to the allocation organization (Eurotransplant) and pathology reports have to be submitted thereafter. In order to assess current procedures we performed a retrospective multicenter analysis in all German transplant centers with focus on accuracy of imaging diagnostic and tumor classification. 1168 primary LT for HCC were conducted between 2007 and 2013 in Germany. Patients inside the Milan, UCSF, and up-to-seven criteria were misclassified with definitive histologic results in 18%, 15%, and 11%, respectively. Patients pretransplant outside the Milan, UCSF, and up-to-seven criteria were otherwise misclassified in 34%, 43%, and 41%. Recurrence-free survival correlated with classification by posttransplant histological report, but not pretransplant imaging diagnostic. Univariate analysis revealed tumor size, vascular invasion, and grading as significant parameters for outcome, while tumor grading was the only parameter persisting by multivariate testing.* Conclusion*. There was a relevant percentage (15-40%) of patients misclassified by imaging diagnosis at a time prior to LI-RADS and guidelines to improve imaging of HCC. Outcome analysis showed a good correlation to histological, in contrast poor correlation to imaging diagnosis, suggesting an adjustment of the LT selection and prioritization criteria.

## 1. Introduction

The best curative approach for HCC in cirrhosis consists of liver transplantation (LT), resulting in elimination of not only the tumor but also the underlying disease. Despite the possible individual patients' benefit of LT, the existing organ shortage forced transplant physicians to establish strict rules of allocation policy that take medical urgency and patient outcome into account. Mazzaferro et al. showed an excellent recurrence-free and overall survival in patients transplanted for small HCCs within the Milan criteria (solitary tumor ≤ 5 cm or up to three tumors ≤ 3 cm) [1, 2]. Thus up to date, especially patients with HCC in liver cirrhosis within the so-called “Milan-Criteria” are selected as potential candidates for LT. Further studies also indicated a comparable good outcome, with a clear individual survival benefit in patients with moderate expansion of the Milan criteria (UCSF criteria or up-to-seven criteria) [3, 4].

In Germany, since the implementation of the Model of End Stage Liver Disease (MELD) score allocation system in December 2006, there has been an exceptional MELD for patients with HCC fulfilling the Milan criteria. Patients receive an assigned MELD score according to 15% 3-month mortality with a step-up in 3-month intervals each by 10% 3-month mortality. However, differences in tumor staging (concerning number and size of lesions) between the pretransplant radiological evaluation and the final histopathological findings present a major concern [5]. This results in an unjustifiable preferential treatment of some patients possibly with reduced outcome, while excluding another group of patients with comparable good treatment outcome due to false classification.

The aim of the presented study is to analyze diagnostic errors between pretransplant imaging diagnostic and posttransplant histological examinations regarding the current liver transplantation program in Germany. Special focus is given to the classification of patients in or outside the Milan criteria due to the relevance for liver allocation and further the extended UCSF and up-to-seven criteria. In a second part, we focus on the outcome and the impact of pretransplant diagnostic and misclassification.

## 2. Methods

The publication describes the results of the retrospective multicenter trial entitled “Retrospective analysis of the current situation of liver transplantation for HCC in Germany with special regard to pre- and posttransplant tumour staging” registered under ELIAC study 2014.02. All patients with primary LT for HCC at a German transplant center between January 01, 2007, and December 31^st^, 2013, were included in the study including a complete one-year survival until December 31^st^ 2014. Data collection was performed based on the available Eurotransplant database. An additional survey was sent to all German liver transplant centers; this voluntary assessment was supported by 12 out of 24 German liver transplant centers. Data included donor/graft data, patient characteristics, tumor data, and patient and tumor follow-up.

During the study period, there was no strict regulation in Germany concerning the pretransplant imaging diagnostic. Usually a cross-sectional imaging with MRI or CT scan was performed; however there were no standards for performing the cross-sectional imaging. Interpretation of the imaging was at the discretion of the reporting doctor, the treating transplant physician, or transplant surgeon. All data were recorded in a completely anonymized database. Overall, 1168 patients/liver transplantations are included in the study.

### 2.1. Statistical Analyzes

The statistical analyses were performed using IBM SPSS software (version 22; IBM Inc, Munich, Germany). A P-value of < 0.05 was considered statistically significant.

Continuous data were expressed as mean/standard deviation; categorical variables were expressed as number/percentage and analyzed by *χ*^2^-test. Survival was assessed by Kaplan-Meier survival curves and Log-rank testing. Subsequently, multivariate analysis using Cox regression model was performed.

## 3. Results

### 3.1. Patient Data

Overall, 1168 patients with primary LT were included for the analysis. Recipients mean age was 57.9 ± 8.4 years. Recipients were predominantly male (n=906; 78%). The leading underlying diagnoses were viral hepatitis (52%) and alcoholic cirrhosis (27%). Five percent of the patients suffered from HCC in noncirrhotic liver. Eighty-three percent of the patients received pretransplant one or more types of bridging therapy. A detailed overview of patients and tumor characteristics is given in Table 1.

### 3.2. Imaging Diagnostic and Histological Examination

The mean number of tumor nodules in the imaging diagnostic was 2.0 ± 2.4 while the mean diameter of the largest tumor nodule was 3.4 ± 2.7 cm. The definitive histological examination revealed a mean of 2.0 ± 2.7 nodules with a mean maximum diameter of 3.1 ± 2.6 cm. In the individual patient the number of tumor nodules was predicted correctly by pretransplant diagnostic in 45.4%, whereas the number was underestimated by one, two, and three or more nodules in 20.9%, 4.1%, and 6.0% while being overestimated by one, two, and three or more nodules in 7.3%, 9.6%, and 6.7%, respectively. In 53.4% of cases the histological examination showed a maximum tumor diameter corresponding to the pretransplant imaging diagnostic (variation ≥ -0.9 - ≤ +0.9 cm). In contrast, the diameter of the largest tumor nodule was underestimated pretransplant by ≥ 1 to < 2 cm, ≥ 2 to < 3 cm, and ≥ 3 cm in 8.9%, 3.0%, and 6.2%, whereas imaging diagnostic overestimated maximum tumor diameter by ≥ 1 to < 2 cm, ≥ 2 to < 3 cm, and ≥ 3 cm in 14.2%, 7.3%, and 6.9%.

Pretransplant being 74%, 81%, and 86% fulfilled the Milan, UCSF, and up-to-seven criteria, respectively. According to posttransplant histological examination 66%, 77%, and 81% of the patients met the Milan, UCSF, or up-to-seven criteria. Every individual patient was analyzed regarding accordance or misclassification inside or outside the criteria depending on posttransplant classification. Patients inside the Milan, UCSF, and up-to-seven criteria were misclassified with definitive histology result outside the mentioned criteria in 18%, 15%, and 11%, respectively. In contrast, patients pretransplant outside the Milan, UCSF, and up-to-seven criteria were posttransplant inside the mentioned criteria in 34%, 43%, and 41%.

Patients without recurrence met the Milan, UCSF, and up-to-seven criteria in 76%, 83%, and 86% based on the pretransplant imaging diagnostic and in comparable percentages based on the posttransplant histology (70%, 83%, and 87%, respectively). As expected, the group of patients with HCC recurrence showed a significant (all P=*0.001*) lower percentage inside the Milan, UCSF, and up-to-seven criteria with pretransplant being 57%, 68%, and 71% and especially posttransplant only being 44%, 57%, and 64% of the patients fulfilling the mentioned criteria.

Subanalysis of patients with or without recurrence revealed a higher rate of misclassification in patients with recurrence (misclassification rates for Milan, UCSF and up-to-seven criteria 13%, 11%, and 8% without recurrence versus 42%, 31%, and 22% with recurrence).

### 3.3. Outcome

Overall, 22% of the patients suffered from HCC recurrence after a mean of 1.9 ± 1.5 years after LT. Survival was initially evaluated using Log-rank test for univariate analyzes and illustrated as Kaplan-Meier survival curves. Patients within the Milan criteria based on the pretransplant imaging diagnostic indicated 1- and 5-year overall patient survival rates of 80.9% and 63.6% and 1- and 5-year recurrence-free survival rates of 76.3% and 59.0%, respectively. Patients fulfilling the UCSF or up-to seven criteria showed comparable recurrence-free survival to patients within the Milan criteria (P=0.833) Likewise, based on the histological findings, comparable recurrence-free survival was shown for patients within the Milan, UCSF, and up-to-seven criteria (P=0.639).

**Figure 1:**
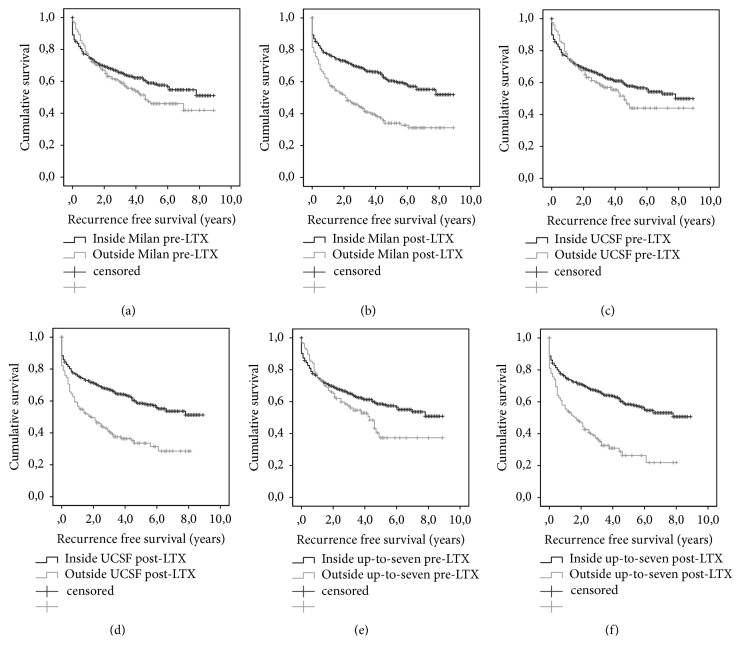
(a + b) Recurrence-free patient survival for patients inside versus outside the Milan criteria based on pretransplant imaging diagnostic (a) or posttransplant histological report (b). (c + d) Recurrence-free patient survival for patients inside versus outside the UCSF criteria based on pretransplant imaging diagnostic (c) or posttransplant histological report (d). (e + f) Recurrence-free patient survival for patients inside versus outside the up-to-seven criteria based on pretransplant imaging diagnostic (e) or posttransplant histological report (f). There was no significant difference in the recurrence-free survival in patients inside versus outside the Milan, UCSF, or up-to-seven criteria based on pretransplant imaging diagnostic (P=0.130; P=0.255; P=0.069). In patients divided by posttranspant histology there was a significant reduced (all P-values 0.001) recurrence-free survival for patient outside the Milan, UCSF, or up-to-seven criteria compared to patients inside the criteria.

Patients outside the Milan, UCSF, and up-to-seven criteria based on posttransplant histological examination showed a highly significant (all P=values=0.001) worse recurrence-free survival compared to patients inside the criteria. Conversely, when preoperative imaging was used, there was a trend towards reduced recurrence-free survival in patients outside the criteria, but the difference was not significant (P-values 0.069-0.255). Figures 1(a)–1(f) show the Kaplan-Meier recurrence-free survival curves for patients inside versus outside the Milan, UCSF, or up-to-seven criteria based on pretransplant (Figures 1(a), 1(c), and 1(e)) or posttransplant (Figures 1(b), 1(d), and 1(f)) diagnostic.

**Figure 2:**
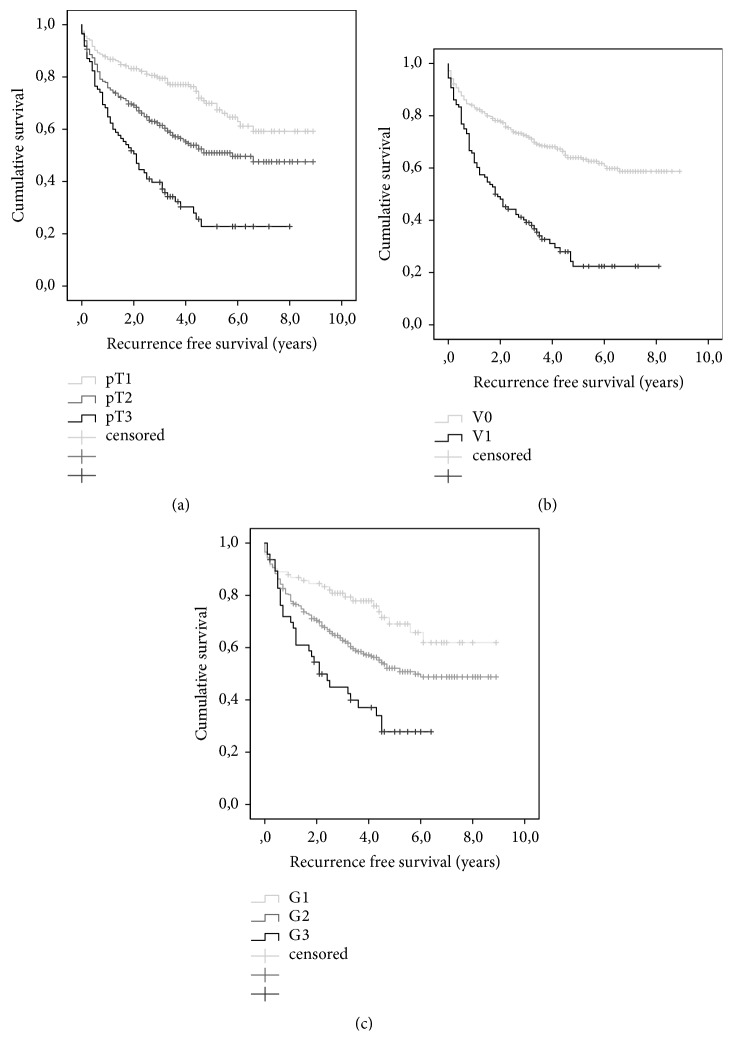
Recurrence-free patient survival in patients depending on primary tumor staging (a), vascular invasion (b), and grading (c). Statistical analysis by Log-rank test showed a significant reduced recurrence-free survival in patients with larger primary tumor stage, microvascular invasion, and higher tumor grading (all P-values= 0.001).

Further outcome analysis was done regarding primary tumor staging (pT1-3; only 6 patients showed a primary tumor stage 4 and were thus excluded), microvascular invasion (V1 versus V0), and tumor grading (G1-3). This showed a significantly reduced recurrence-free survival in patients with high tumor staging, microvascular invasion, and more undifferentiated tumor grading (all P=0.001). The corresponding Kaplan-Meier survival curves are shown in Figures 2(a)–2(c).

**Figure 3:**
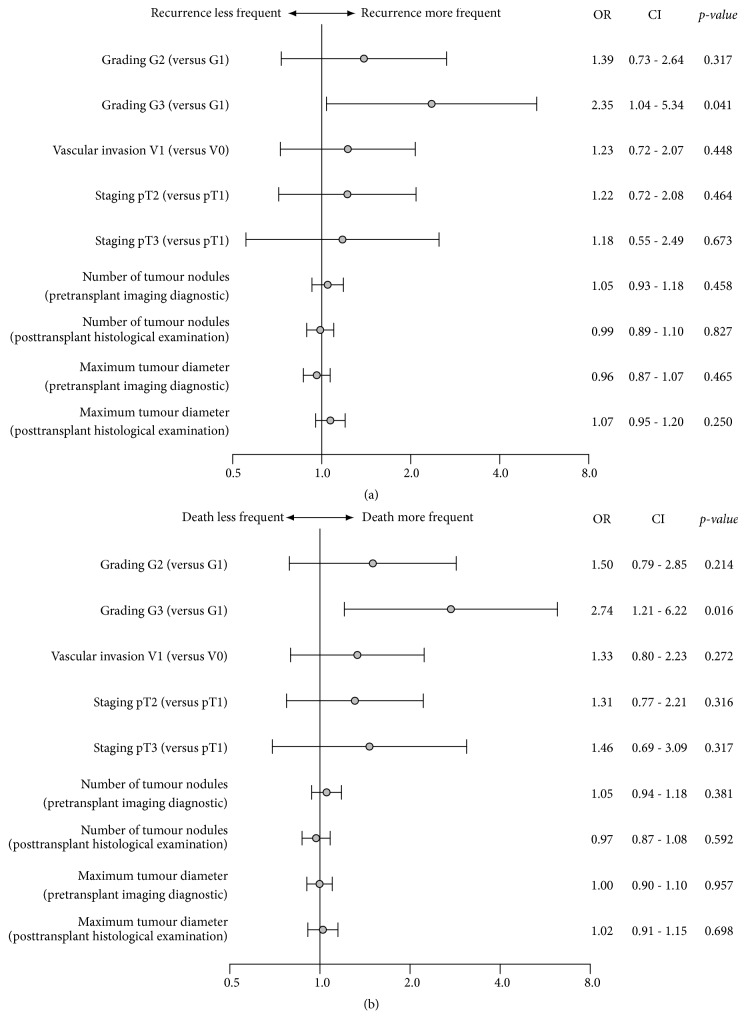
Multivariate Cox regression analysis of recurrence-free patient survival (a) and overall patient survival (b). Multivariate Cox regression analysis regarding overall patient survival and recurrence-free patient survival showed no significant influence of regarding primary tumor stage (pT), microvascular invasion, (V) and number of tumor nodules or maximum tumor diameter pretransplant as well as posttransplant. However, there was a significant elevated risk of patients death and tumor recurrence in patients with undifferentiated tumor grading (hazard ratio 2.353 G3 stage versus G1 stage; P=0.041 for patient survival and hazard ratio 2.739 G3 stage versus G1 stage; P=0.016 for recurrence-free survival).

We performed a multivariate Cox regression analysis with the parameters number of tumor nodules and maximum tumor diameter in cm, based on pretransplant imaging diagnostic as well as posttransplant histological examination, including primary tumor (pT), microvascular invasion (V), and tumor grading (G) regarding patient survival and recurrence-free survival. In the multivariate analysis the parameters number of tumor nodules and maximum tumor diameter pretransplant as well as posttransplant, primary tumor staging and even microvascular invasion failed to reach statistically significance. However, there was a significant high risk of patients death and tumor recurrence with undifferentiated tumor grading (hazard ratio 2.353 G3 stage versus G1 stage; P=0.041 for patient survival and hazard ratio 2.739 G3 stage versus G1 stage; P=0.016 for recurrence-free survival). Forest plot shows the results of multivariate analysis (Figures 3(a) and 3(b)).

## 4. Discussion

In selected patients with early stage HCC and liver cirrhosis, LT offers the best curative option. In general, HCC diagnosis in cirrhotic patients is based on imaging diagnostic without further need of tumor biopsy. In Germany, 2006, the MELD score allocation system was implemented, including a privilege for patients with HCC fulfill the Milan criteria. In the past, including the study period, the German waitlist guidelines did neither define radiologic imaging nor who shall confirm the results. Hence it was at the discretion of surgeons or physicians to decide if a HCC was within the Milan criteria. An issue raised attention and much criticism during the audits of the liver transplant centers by the Permanent Committee for Organ Transplantation of the German Medical Association [6–8]. In order to assess current procedures we performed a retrospective multicenter analysis in all German transplant centers with focus on the accuracy of the pretransplant imaging to identify misclassification and resulting allocation failure as well as its impact on clinical outcome.

In our study we found an expected difference between the results of the imaging diagnostic and the real tumor load confirmed by the explanted liver graft. Both the number of tumor nodules and the diameter of the largest node were predicted correctly only in about 50% of the patients. In the other half of the patients, the tumor number had underrated or overrated one or more nodes or the maximum diameter of the tumor had under- or overestimated more than 1 cm. Already Yao et al. analyzed 70 LTX for HCC, thereof the tumor size was correctly estimated by ultrasound, CT scan, or MRI in only 62.5 to 80.8% of the patients and the number of nodules was correctly predicted in only 25 to 50% [3].

Regarding the correct classification of the individual patient, we found for patients inside the Milan, UCSF, and up-to-seven criteria based on pretransplant imaging diagnostic a discrepancy in the histological examination of 18%, 15%, and 11%, respectively. Mazzaferro et al. already reported in his earlier publication defining LT as standard procedure for HCC within the Milan criteria a difference of 27% between pretransplant staging and posttransplant histology [2]. Another single center study regarding the correct classification of patient with HCC found comparable results with pretransplant misclassification of 25% and 26% of the patients inside the Milan or UCSF criteria [9]. To our understanding, this is the first study where patients from a national cohort reported to the allocation organization (Eurotransplant) for waitlist registration on a national waitlist are evaluated based on the documented imaging as well as the pathology report. Audition of the German liver transplantation program indicated serious problems regarding HCC diagnosis and resulting liver allocation.

Actually, according to the guidelines of the EASL-EORTC (European Association for the Study of the Liver, European Organization for Research and Treatment of Cancer) and the AASLD (American Association for the Study of Liver Diseases) a contrast enhanced CT or MRI, showing the HCC typical hallmarks of arterial uptake, followed by venous/late phase washout, is the standard for diagnostic [10, 11]. The newly developed Liver Imaging Reporting and Data System (LI-RADS) follows a similar approach. The LI-RADS was developed based on extensive literature review and a multidisciplinary committee of diagnostic and interventional radiologists, hepatologists, liver transplant and hepatobiliary surgeons, pathologists, and informatics enables classification of each liver lesion into five categories from definitively benign to definitively HCC. The criteria include, in addition to arterial phase hyperenhancement and washout, the observation diameter, the threshold growth, and the capsule appearance.

Based on the preliminary findings of the audition group, a change of the guidelines for exceptional MELD in HCC patients was already performed in 2016 in Germany. This included a change towards the UNOS T2 system with clearly defined kind of imaging diagnostic. For HCC diagnosis evidence of arterial hypervascularization with contrast agent washout in a 3-phase (late-arterial, portal venous, and late phase) cross-sectional imaging diagnostic is mandatory. Liver lesions between 1 cm and < 2 cm need 2 contrast enhanced procedures (MRI, CT scan or contrast enhanced ultrasound); for tumors > 2 cm 1 contrast enhanced procedure (MRI or CT scan) is sufficient. For the assessment of the HCC stage, a standardized report with a formal certification of the imaging results by a board certified radiologist is required. Additionally, since 2013 an interdisciplinary and organ-specific Transplantation Conference is mandatory to confirm evaluation findings and include a patient in the waiting list [12].

According to the German Transplant Legislation also outcome is a pivotal element for the development of guidelines. During the present study special analysis of our patients with HCC recurrence revealed a clearly higher rate of misclassification with upstaging outside the Milan, UCSF, or up-to seven criteria based on histological examination varying from 22 to 42%.

Typically, the number of patients undergoing LT classified outside the Milan criteria and especially outside more extensive, e.g., UCSF or up-to seven, criteria based on pretransplant imaging diagnostic is low. 26%, 19%, and 14% of the patients were graded pretransplant outside the Milan, UCSF, and up-to-seven criteria. In these patients hepatectomy histology revealed a misclassification in 34%, 43%, and 41%, respectively. These data demonstrate especially in the patient group staged pretransplant outside more extensive criteria the proportion of misclassified patients was increasing, corresponding to almost 50% incorrect results for patients pretransplant outside the UCSF or up-to-seven criteria. However, our data regard only a subgroup of patients with performed LT and therefore a certainly not representative cohort. Nevertheless, we should be aware of the wrong overestimation of HCC load in imaging diagnostic with resulting exclusion of patients from the LT option or at least the exclusion of extra points.

Outcome analysis in patients showed an overall survival of 80.9% and 63.6%, while recurrence-free survival was 76.3% and 59.0% in 1 and 5 years, respectively, within the Milan criteria based on pretransplant imaging diagnostic. These data are comparable to the literature and ELTR data indicating 1-year-survival rates between 80% and 90% and 5-year survival rates varying from 50% to 80% in patients undergoing LT for HCC [13, 14]. In this study, we found no significant difference in the recurrence-free survival in patients inside the Milan criteria versus patients inside the UCSF or up-to-seven criteria based on imaging diagnostic. Likewise, the outcome was comparable for patients inside the Milan, UCSF, or up-to-seven criteria, if staging was based on histological examination. This is in concordance with previous data proving a comparable outcome in patients beyond the Milan criteria but within the UCSF or up-to-seven criteria [3, 4]. The outcome between patients inside versus outside the Milan or UCSF criteria has already been analyzed in a number of studies with a clear survival benefit in patients fulfilling the criteria [15, 16]. In our study outcome analysis showed significantly better recurrence-free survival in patients inside versus outside the Milan, UCSF, and up-to-seven criteria if classification was based on histological report (all: P=0.001). In contrast, no significant differences were noticed if patients were classified by pretransplant imaging diagnostic (P-values=0.069-0.255). However, it remains to be considered that patients within the Milan criteria receive an advantage in organ allocation, while patients outside the Milan criteria may have a longer waiting time or have organs allocated as so-called rescue allocation (organs which are not accepted in primary allocation).

Univariate analysis between tumor histology and outcome revealed a significant impact of high primary tumor stage, vascular invasion, and poor tumor differentiation, resulting in clearly decreased recurrence-free survival. In addition, the multivariate analysis remained only with undifferentiated tumor grading significant with regard to the outcome, whereas there was no significant influence of vascular invasion, primary tumor staging or the number of tumor nodules, respectively, or maximum tumor diameter neither in the pretransplant nor in the posttransplant diagnostic. These data are in accordance with other studies indicating a significant influence of poorly differentiated HCC on recurrence [17–19]. Likewise, the Toronto group recommended the appropriate impact of poorly differentiated HCC; therefore patients with advanced tumor (no restrictions on tumor size or number, only exclusion of extrahepatic disease) were considered for LT in case of good or moderate differentiated tumor, after exclusion of poorly differentiated HCC by pretransplant biopsy. Outcome analysis illustrated a comparable patient survival and recurrence risk within this extended criteria group compared to standard group within the Milan criteria without requiring preoperative biopsy [20, 21]. However, pretransplant liver biopsy takes the risk of relevant complications (bleeding, tumor seeding). Additionally, a recent study could also show a poor concordance (*κ*=0.22) of preoperative needle biopsy to final explant pathology.

In summary, the accuracy of pretransplant imaging diagnostic in the analyzed patients was nonsatisfying, with an underestimation of HCC in 11-18% patients pretransplant fulfilling the criteria and an overestimation of the tumor in 34-43% of the patients pretransplant outside the criteria. Only patients before transplant stage within the Milan criteria were given privilege; however, our data suggest, consistent with previous data, that even patients without the Milan criteria but inside UCSF or up-to-seven criteria could undergo LT with a comparable outcome.

Outcome analysis showed a good correlation to histological classification and in contrast a poor correlation to imaging diagnosis, whereas the privilege is associated with the imaging diagnosis. Likewise there was a significant influence of tumor grading on patient survival and HCC recurrence. Further pretransplant diagnostic could help to select patients with good tumor biology for LT (e.g., biopsy with resulting tumor grading); however the reliability is limited.

The presented results demonstrate the necessity of optimizing particular the imaging and documentation in the study period in Germany. As a result a change towards the UNOS T2 system as well as a clear definition of diagnostic imaging and formal certification of the results by a board certified radiologist was regulated by law in 2016.

Nevertheless, it opens the question of what we are missing. Larger than Milan, UCSF, or up-to-seven tumors had good outcomes in selected patients and also there were patients within the above mentioned criteria that did not. One of the results pointing into the direction of tumor biology is the grading. Since pretransplant tumor biopsies are rather random in their results, the question remains as to how to individualize the diagnosis. In order to proceed with a national study including all German transplant centers has been established and will soon start hopefully filling this gape of information.

## Figures and Tables

**Table 1:** Patient and tumor characteristics.

Recipient age (years);	57.9 ± 8.4
mean ± standard deviation	

Recipient gender (male/female [%])	78% / 22%

Diagnosis [%]	
(i) Viral hepatitis	52%
(ii) Alcoholic cirrhosis	27%
(iii) Autoimmune hepatitis/PBC/PSC	3%
(iv) Unclear cirrhosis	6%
(v) Other cause	5%
(vi) HCC in non-cirrhotic liver	5%

Bridging therapy [%]	
(i) Resection	21%
(ii) TACE	75%
(iii) RFA	18%
(iv) Ethanol injection	9%
(v) Radiation	6%
(vi) Cryotherapy	<1%
*∗* >100% due to patients with multiple bridging therapies	

Tumor staging [%]	
Primary tumor	
(i) T1	39%
(ii) T2	45%
(iii) T3	16%
(iv) T4	1%
Regional lymph nodes	
(i) N0	98%
(ii) N1	2%
Distant metastasis	
(i) M0	99%
(ii) M1	1%
Vascular invasion	
(i) V0	79%
(ii) V1	21%
Invasion lymphatic vessels	
(i) L0	94%
(ii) L1	6%

Resection boundaries [%]	
(i) R0	99%
(ii) R1	1%

Grading [%]	
(i) G1	17%
(ii) G2	73%
(iii) G3	11%

## Data Availability

The data used to support the findings of this study are included within the article.
